# Editorial: Advances in the microbiome, immunity and cancer interplay

**DOI:** 10.3389/fcimb.2025.1568679

**Published:** 2025-03-31

**Authors:** Gratiela Gradisteanu Pircalabioru, Núria Mulet Margalef

**Affiliations:** ^1^ Department of Botany and Microbiology, Faculty of Biology, University of Bucharest, Badalona, Spain; ^2^ Research Institute of University of Bucharest, Badalona, Spain; ^3^ Centre of Excellence in Bioengineering, National University of Science and Technology Politehnica Bucharest, Badalona, Spain; ^4^ Medical Oncology Department, Catalan Institute of Oncology, Badalona, Spain; ^5^ Institute for Health Science Research Germans Trias i Pujol (IGTP), Badalona, Spain

**Keywords:** microbiome & dysbiosis, microbiome, cancer, cancer immunology, omics

The intricate interplay between the human microbiome and cancer immunity is a rapidly expanding field, poised to revolutionize oncological therapeutic strategies (Shi et al., 2024). This dynamic area of research illustrates that the microbiome functions not merely as a passive entity but as an active participant in modulating immune responses, influencing the progression of diseases like colorectal cancer (CRC) and other malignancies, and shaping the efficacy of treatments.

CRC is one of the most prevalent cancers globally, and its traditional treatment protocols—which typically include surgery, chemotherapy, and radiotherapy—have recently been enhanced by the integration of immune checkpoint blockers (ICBs) mainly in the management of MSI-H/dMMR colorectal cancer (Morgan et al., 2023). These advancements underscore the microbiome’s pivotal role in modulating treatment responses. For instance, emerging evidence suggests that specific microbiota compositions can significantly influence the toxicity and efficacy of both radiotherapy and ICBs. Fecal microbiota transplantation (FMT) is being explored to mitigate these adverse effects and improve therapeutic outcomes, highlighting a novel approach in CRC treatment protocols. The review manuscript by Van Dingenen et al. presents the current preclinical and clinical data on CRC treatments, focusing on the interaction between the gut microbiome and the toxicity and efficacy of radio- and immunotherapies, and examines the potential benefits of incorporating FMT into treatment protocols (Frontiers | *Dissecting the role of the gut microbiome and fecal microbiota transplantation in radio- and immunotherapy treatment of colorectal cancer*).

The microbiota is notably altered in CRC patients, not only in tumor tissue (compared with surrounding healthy mucosa) and feces, but also changes in their oral microbiota have been evidenced. The study by Wang et al. focuses on assessing levels of immunoglobulin SIgA, inflammatory factors, and lymphocyte subsets in intestinal mucosa at varying intervals post-radiotherapy, aiming to determine the optimal timing for surgery post-radiotherapy and explore potential probiotics or immunomodulators through high-throughput sequencing of bacterial 16s rRNA in saliva microbiota (Frontiers | *The recovery of intestinal barrier function and changes in oral microbiota after radiation therapy injury*).

Moreover, the role of the microbiome extends beyond CRC to other cancers, such as gastric cancer (GC) and breast cancer. In GC, Mendelian randomization studies have pinpointed specific gut microbiota that potentially affect cancer risk, suggesting new prevention and treatment avenues (Zhang et al., 2024). The analysis by Li et al. confirmed negative and positive associations between specific gut microbiota taxa and GC risk, and identified several blood metabolites associated with decreased GC risk. Notably, traits such as lipids and phospholipids in different lipoprotein particles were implicated as mediators in the relationship between certain gut microbiota taxa and GC risk, with modest mediation proportions suggesting indirect influence (Frontiers | *Exploring the mediating role of blood metabolites in the relationship between gut microbiota and gastric cancer risk: a Mendelian randomization study*). In breast cancer, the research by Zeber-Lubecka et al. indicated that menopausal status may influence microbiome profiles, potentially affecting disease outcomes and responsiveness to treatment (Frontiers | *Breast cancer but not the menopausal status is associated with small changes of the gut microbiota*).

Additionally, *Helicobacter pylori*, a bacterium linked to gastric cancer, also impacts on extragastric conditions like CRC. By altering the microbial communities not only in the stomach but also in the distal large intestine, *H. pylori* infection modifies intestinal immunity and microbiome signatures, potentially contributing to tumor development in the colon. The review by Engelsberger et al. focuses on the complex interplay between *H. pylori* infection, intestinal microbiota changes, and intestinal immunity, as well as the effects of antibiotic eradication therapy on these dynamics (Frontiers | *Effects of Helicobacter pylori infection on intestinal microbiota, immunity and colorectal cancer risk*).

In non-small cell lung cancer (NSCLC), the integration of Traditional Chinese Medicine (TCM) syndrome classifications and microbiota profiling has opened new avenues for enhancing the efficacy of immunotherapy treatments. This approach exemplifies the potential of leveraging microbiome insights to refine treatment strategies across various cancer types. The study by Li et al. categorized advanced NSCLC patients based on TCM syndromes and treated them with PD-1 inhibitors and involved analyzing stool and blood samples before and after treatment using 16S rRNA sequencing and flow cytometry to assess microbial changes and immune cell dynamics (Frontiers | *Exploring the relationship between intestinal microbiota and immune checkpoint inhibitors in the treatment of nonsmall cell lung cancer: insights from the “lung and large intestine stand in exterior-interior relationship” theory*). Results showed that patients with spleen-lung Qi deficiency and Qi-Yin deficiency syndromes responded better to immunotherapy. Significant microbial shifts were observed in strains of Clostridia, Lachnospiraceae, and Lachnospirales. Patients with positive PD-L1 expression showed improved immune profiles, including increased ratios of CD3+%, CD4+%, and CD4+/CD8+ T cells and reduced suppressor cell subsets. The findings suggest that specific microbial strains can serve as biomarkers for NSCLC therapy outcomes, with TCM classifications providing a valuable framework for predicting and enhancing immunotherapy efficacy.

Wang et al. investigated the causal relationship between gut microbiome composition and biliary tract cancer (BTC), aiming to evaluate the microbiome’s utility in early BTC diagnosis (Frontiers | *The causal relationship between gut microbiota and biliary tract cancer: comprehensive bidirectional Mendelian randomization analysis*). Utilizing data from the Biobank Japan (BBJ) database, which included 418 BTC cases and 159,201 controls, and gut microbiota data from 18,340 participants in the MiBioGen consortium, the study employed Inverse Variance Weighting (IVW) for analysis. Certain microbiota members, such as the Streptococcaceae, Veillonellaceae, and Dorea, had a protective effect against BTC, while Lentisphaeria, Lachnospiraceae FCS020 Group, and Victivallales were associated with increased BTC risk. The reverse causal analysis, using BTC as the exposure, showed associations between BTC and various microbiota types. These findings suggest beneficial or detrimental causal relationships between specific gut microbiota and BTC risk, offering potential insights for developing targeted therapeutic strategies for BTC prevention and improving patient outcomes.

The future of microbiome research in cancer therapy is likely to focus on integrating multi-omic data (genomics, transcriptomics, metabolomics and immune response, among others) to better understand the host-microbiome interactions at play. This could lead to the development of more precise biomarkers for predicting treatment response and more targeted therapies that specifically modify the microbiome to improve patient outcomes.

As we continue to decipher the complex interactions between the microbiome and the immune system in the context of cancer, it becomes increasingly clear that the microbiome holds significant untapped therapeutic potential. The integration of microbiome manipulation into cancer treatment regimens offers a promising avenue to enhance the effectiveness of existing therapies and potentially overcome resistance to treatment. The next decade of research will be crucial in moving these concepts from bench to bedside, offering new hope for patients facing cancer.
